# Use and the Users of a Patient Portal: Cross-Sectional Study

**DOI:** 10.2196/jmir.9418

**Published:** 2018-09-17

**Authors:** Bas Hoogenbosch, Jeroen Postma, Janneke M de Man-van Ginkel, Nicole AM Tiemessen, Johannes JM van Delden, Harmieke van Os-Medendorp

**Affiliations:** 1 Department of Information Technology University Medical Centre Utrecht Utrecht Netherlands; 2 Erasmus School of Health Policy and Management Erasmus University Rotterdam Rotterdam Netherlands; 3 Julius Centre for Health Sciences and Primary Care University Medical Centre Utrecht Utrecht Netherlands; 4 Department of Dermatology and Allergology University Medical Centre Utrecht Utrecht Netherlands

**Keywords:** patient portals, eHealth literacy, Unified Theory of Acceptance and Use of Technology

## Abstract

**Background:**

Patient portals offer patients access to their medical information and tools to communicate with health care providers. It has been shown that patient portals have the potential to positively impact health outcomes and efficiency of health care. It is therefore important that health care organizations identify the patients who use or do not use the patient portal and explore the reasons in either case. The Unified Theory of Acceptance and Use of Technology (UTAUT) is a frequently used theory for explaining the use of information technology. It consists of the following constructs: performance expectancy, effort expectancy, social influence, facilitating conditions, and behavioral intention to use.

**Objective:**

This study aimed to explore the prevalence of patient portal use and the characteristics of patients who use or do not use a patient portal. The main constructs of UTAUT, together with demographics and disease- and care-related characteristics, have been measured to explore the predictive factors of portal use.

**Methods:**

A cross-sectional study was conducted in the outpatient departments for adult patients of a university hospital in the Netherlands. Following outcomes were included: self-reported portal use, characteristics of users such as demographics, disease- and care-related data, eHealth literacy (modified score), and scores of UTAUT constructs. Descriptive analyses and univariate and multivariate logistic regression were also conducted.

**Results:**

In the analysis, 439 adult patients were included. Furthermore, 32.1% (141/439) identified as being a user of the patient portal; 31.2% (137/439) indicated as nonusers, but being aware of the existence of the portal; and 36.6% (161/439) as being nonusers not aware of the existence of the portal. In the entire study population, the factors of being chronically ill (odds ratio, OR 1.62, 95% CI 1.04-2.52) and eHealth literacy (modified score; OR 1.12, 95% CI 1.07-1.18) best predicted portal use. In users and nonusers who were aware of the portal, UTAUT constructs were added to the multivariate logistic regression, with chronically ill and modified eHealth literacy sum score. Effort expectancy (OR 13.02, 95% CI 5.68-29.87) and performance expectancy (OR 2.84, 95% CI 1.65-4.90) are shown to significantly influence portal use in this group.

**Conclusions:**

Approximately one-third of the patients of a university hospital self-reported using the patient portal; most expressed satisfaction. At first sight, being chronically ill and higher scores on the modified eHealth literacy scale explained portal use. Adding UTAUT constructs to the model revealed that effort expectancy (ease of use and knowledge and skills related to portal use) and performance expectancy (perceived usefulness) influenced portal use. Interventions to improve awareness of the portal and eHealth literacy skills of patients and further integration of the patient portal in usual face-to-face care are needed to increase use and potential subsequent patient benefits.

## Introduction

### Background

**Figure 1:**
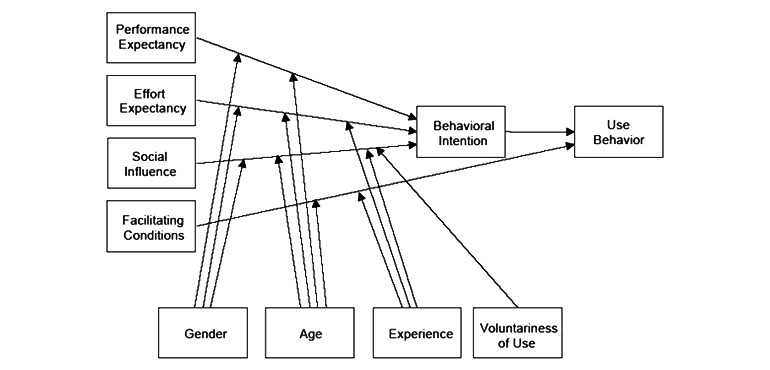
The Unified Theory of Acceptance and Use of Technology (UTAUT)—copied with permission from Venkatesh et al [24].

eHealth is defined as the use of information and communication technologies for health [1]. It is known that use of eHealth can lead to improved care for chronically ill patients [2,3]. Moreover, health policy supports the benefits of eHealth—mainly because eHealth can lead to a decrease in the information asymmetry between the health care provider and patient by facilitating access to general and personal medical information [4]. A patient portal is a form of eHealth. The medical dictionary defines a patient portal as “A domain in an electronic health record that allows patients to access their records or communicate with their health care providers” [5]. The types of patient portals vary between health care institutions; however, in most portals, patients have access to their medical information and are able to use tools to exchange information and to communicate electronically with the health care provider in a secure manner [6-8]. Patient portals have the potential to increase patient engagement in health care [9]. Research has shown several benefits that can be linked to the introduction of a patient portal [9,10]. First, the use of a patient portal could lead to better clinical outcomes, for instance, diabetes measures [11,12]. Second, associations have been found between the use and availability of patient portals and better communication between the patient and health care provider, quality of care [11,13], improved self-management, and a higher level of patient satisfaction [13]. Third, there is evidence that follow-up care of patients with atopic dermatitis by using a patient portal leads to a substantial cost reduction in the follow-up care of patients with atopic dermatitis, mainly through a reduction of work absenteeism [14]. However, lower health care consumption by the use of patient portals could not be validated [15]. The review by Otte-Trojel showed that health care consumption increased in 5 out of 8 studies regarding health care consumption and patient portal implementation. Two studies found no change, and in one study, lower health care use was reported. It was therefore concluded that patient portals were used in addition to usual care rather than as a replacement [15]. Conversely, a systematic review reported that there is mixed evidence about the effects of patient portals on outcomes and satisfaction. Furthermore, differences in study methodology and portal functionalities limit comparison and generalizability of results [16]. However, the success of patient portals and the subsequent achievement of the aforementioned effects are intrinsically linked to the extent to which they are used [17]. A systematic review indicated that portal use is influenced by factors such as age, educational level, ethnicity, and health literacy. In addition, provider endorsement, communication tactics, the ease of use of a portal, the relative advantage of a portal, and the observability of the benefits of the portal transpired to have a positive influence on portal use [9,18,19]. Different models explain the use and adoption of information technology in health care, but the technology acceptance model (TAM) [20] is commonly used [21]. According to this model, intention to use and use of technology is influenced by perceived usefulness and ease of use. A study by Noblin et al [22] used the TAM to investigate the intention to use a personal health record and showed that the decision of patients to adopt a personal health record was influenced by perceived usefulness and technology barriers (perceived ease of use). An extension of TAM is The Unified Theory of Acceptance and Use of Technology (UTAUT) [23], which is also based on the Motivational Model, the Model of Personal Computer Use, the Theory of Diffusion of Innovations, and the Social Cognitive Theory. This is depicted in Figure 1 [24]. The figure, developed by Venkatesh et al [24], shows that technology acceptance (*use behavior*) is dependent on the intention to use it (*behavioral intention*) and the conditions that facilitate the use (*facilitating conditions*). Furthermore, it shows that the intention to use a new technology is the result of the usefulness (*performance expectancy*) and the ease of use (*effort expectancy*) of the new technology. In addition, the social environment (*social influence*) has an effect on the intention to use a new technology.

The external variables that influence the mechanism of the UTAUT are gender, age, experience, and voluntariness of use. Generally, young men score higher on performance expectancy; young women with little technological experience score higher on effort expectancy; older women with little technological experience and in a situation in which use is voluntary, score higher on social influence; and older people with more technological experience score higher on facilitating conditions [24]. UTAUT has been empirically validated.

Venkatesh et al [24] showed that the UTAUT can explain 70% of the variance in usage intention, which suggests that the UTAUT is a good predictor of the ultimate likelihood to use a new technology. The UTAUT provides a reliable prediction of the use of technology at 152 German companies [25], the computer use frequency at a Belgian university [26], and the adoption of social media at 409 nonprofit organizations in the United States [27]. UTAUT has also been used previously in health care. For example, a study by Kim et al [28] showed that the acceptance of a mobile electronic medical record was influenced by performance expectancy and attitude.

### Aim

The aim of this study was to explore the prevalence of patient portal use and the characteristics of patients who use or do not use a patient portal. The main UTAUT constructs—performance expectancy, effort expectancy, social influence, facilitating conditions, and behavioral intention to use—together with demographics and disease- and care-related characteristics were measured to explore predicting factors of portal use.

## Methods

### Study Design

A cross-sectional study was conducted in the outpatient departments of the University Medical Centre Utrecht (UMCU), the Netherlands. Since 2015, all UMCU patients have had real-time access to a patient portal containing the following functionalities: insight into the medical file with reports of consultations and diagnostic results; tools such as questionnaires and diaries; viewing appointments; sending and receiving e-consultation (defined as a secure message for patient-provider communication within the patient portal); and adding personal information. The patient portal of the UMCU was provided by Chipsoft, a software company, and integrated into Hix, the electronic medical file.

### Setting and Subjects

The research population consisted of adult patients visiting one of the outpatient departments of the UMCU in April 2016. Fluency in Dutch (speaking and reading) was an inclusion criterion, as the questionnaire and information in the medical file were in Dutch. Children (under the age of 18 years) and inpatients were excluded.

Patients visiting a specific outpatient department for functional diagnostics—for example, electrocardiogram, lung function tests, or colonoscopy—were also excluded to prevent double counting because these patients may already have visited a medical specialist at an outpatient department. To obtain a sample that is in proportion with the size of the different outpatient departments, the ratio of the number of outpatients in a department to the total number of patients as a whole was calculated for each department. This process led to the following distribution: internal outpatient clinics (123/398; 30.9%), surgery (174/398; 43.7%), neurology or psychiatry (42/398; 10.6%), cardiology and lung diseases (41/398; 10.3%), woman and baby department (17/398; 4.3%), and genetics (1/398; 0.3%). We aimed to include about 398 patients by convenience sampling. We recruited about 500 patients because of anticipated incomplete data of about 20% (100/500).

Before the commencement of data collection, the Medical Ethics Review Committee (MERC) of the UMCU declared that the Medical Research Involving Human Subjects Act (WMO) did not apply to this study (MERC protocol number 16/170C) and therefore an official approval of this study by the MERC UMCU was not required under the WMO.

### Data Collection and Outcomes

Two researchers, both wearing UMCU t-shirts, introduced themselves in the outpatient clinic and asked patients who were waiting whether they were ready to participate in the study. Data were collected using a structured paper questionnaire. The questionnaire commenced with an information letter, which explained that by completing the questionnaire, patients gave permission to use their data for this research project. This was undertaken to indicate informed consent.

The main outcome of the study was the patient-reported use of the patient portal, which had been operationalized by asking the patients whether they had used the patient portal or not. To obtain more background on usage, the patients were asked which functionalities of the patient portal they used and whether they were satisfied with the patient portal. With regard to the presumed gap between intention and behavior [29], patients were also asked to report whether they intended to use the patient portal in the future.

Secondary outcomes regarding characteristics of users were demographics, disease- and care-related data, and eHealth literacy. Demographic data consisted of gender; age; level of education (low: no education or low secondary or vocational education, intermediate: intermediate vocational education or higher general secondary education, high: higher vocational education or university); travel distance (estimated travel time in minutes); and life status ([not] working, studying, or retired). Background of being native Dutch was operationalized by asking the country of birth of the person and his or her parents. According to the definition of Statistics Netherlands, if both parents were born in the Netherlands, a person is native Dutch. If at least one of the parents was not born in the Netherlands, a person is not native Dutch [30]. Disease- and care-related data were also collected. This consisted of satisfaction with care (using a 5-point Likert scale ranging from *strongly satisfied* to *strongly dissatisfied*) and self-reported chronic illness (operationalized by presenting a short definition and examples of chronic diseases and then asking the patient whether he or she was a chronic patient). eHealth literacy data were collected using the Dutch translation of the eHealth literacy questionnaire from the study by Van der Vaart et al [31]. Reliability of the original English version [32] and Dutch translation [31] was adequate. Patients were asked to indicate to what extent they agreed or disagreed with the given statements on a 5-point Likert scale. The Likert scale was considered to be a continuous scale. The numbers were added up to compute a sum score. Total scores ranged from 7 to 35, with higher scores representing higher self-perceived eHealth literacy. The eHealth literacy questionnaire consisted of 8 questions, but 1 question “I know how to use the health information I find on the internet to help me” was accidentally excluded. We, therefore, computed a modified sum score over the other 7 questions. To maximize transparency, we also reported scores on the individual questions. The internal consistency (Cronbach alpha) of the 7 questions was .929.

UTAUT constructs were measured using questions about performance expectancy (3 questions about usefulness of the portal for health and care; Cronbach alpha=.91), effort expectancy (6 questions about ease of use and knowledge and skills required to use the portal; Cronbach alpha=.89), social influence (2 questions about influence of health care professionals and loved ones or relatives; Cronbach alpha=.45), facilitating conditions (3 questions about available help and information; Cronbach alpha=.58), behavioral intention to use (1 question), and recommendation to others (1 question, considered as satisfaction with the portal), according to the operationalizations constructed in the study by Kohnke et al [33]. Patients gave their answers on statements, with a 5-point Likert scale ranging from strongly disagree, neutral to strongly agree as well as the answer option “no idea/not applicable.”

### Data Analysis

Analyses were conducted with IBM SPSS Statistics, version 21 (Armork, New York, USA), in 3 groups.

#### Analyses in the Total Group

Descriptive statistics were used to analyze the users, nonusers aware of the portal, and nonusers not aware of the portal. A chi-square test was used to test whether there were significant differences between the 3 groups on the categorical variables. The continuous variables with a normal distribution were compared using analysis of variance (ANOVA; 3 groups) or an independent *t* test. A *P* value <.05 was considered to be significant.

Logistic regression was used to explain the use of the patient portal. The use of the patient portal was used as a dependent variable. Gender, age, high or intermediate educational level (vs low educational level), working (vs not working, retired, or studying), travel time, chronic patient, and the modified eHealth literacy sum score were used as independent variables. First, univariate logistic regression analyses were performed. Predictors with *P*<.20 [34] were included in the final multiple logistic regression model, using the *Enter* method. Predictors with *P* ≤.05 were considered to contribute significantly to the prediction of the use of the patient portal.

#### Analyses in Users and Nonusers Who Were Aware of the Portal

Frequency scores of the UTAUT constructs were collated, and percentages on strongly disagree, neutral, strongly agree, and not applicable or no opinion, per mechanism, were calculated. In addition, mean scores of UTAUT constructs (except recommendation) were computed, and scores of patients who used the portal and of nonusers aware of the existence of the portal were compared. Subsequently, a second multivariate logistic regression was conducted for users and those nonusers aware of the existence of the portal, including significant predictors from the first set of analyses in the total group. To analyze the predicted value of UTAUT constructs, univariate analyses were conducted with these constructs, and the constructs with a *P* value<.20 were included in the third multivariate logistic regression together with significant predictors from the first analyses in the total group.

## Results

### Response and Sample Characteristics

A total of 513 patients were willing to participate in this study. Of them, 74 (74/513, 14.4%) patients were excluded because the main question about portal use was not answered or their age was either below 18 years or unknown. In total, 439 patients were included in the analyses. The mean age was 53.0 years (SD 17.4, range 18-88 years); 51.2% (225/439) were females. Patients visited different outpatient hospital departments: 34.4% (151/439) visited the internal outpatient clinics, 27.1% (119/439) surgery, 11.6% (51/439) neurology or psychiatry, 8.0% (35/439) cardiology and lung diseases, 4.3% (19/439) department of woman and baby, and 0.5% (2/439) genetics. In 14.1% (62/439) cases, the information regarding the consulting department was missing.

### Portal Use and Satisfaction

In this study, 32.1% (141/439) of the patients indicated being users of the patient portal; 31.2% (137/439) indicated being nonusers of the patient portal, but being aware of the existence of the portal; and 36.7% (161/439) indicated being nonusers and not being aware of the existence of the portal. Portal users, compared with nonusers, were significantly younger, less often retired, more often native Dutch, more often chronically ill, and more often very satisfied with hospital care. In addition, portal users scored higher on the modified eHealth literacy scale (Tables 1 and 2).

**Table 1:** Differences between portal users and nonusers.

Characteristics		User (n=141)	Nonuser but aware of portal (n=137)	Nonuser not aware of portal (n=161)	*P* value for differences
Travel time in minutes (missing n=2), mean (SD)		43 (47)	38 (24)	38 (26)	.33^a^
Age in years, mean (SD)		50 (15)	53 (17)	55 (19)	.02^a^
**Gender (missing n=1), n (%)**					.48^b^
	Man	63 (44.7)	67 (49.3)	83 (51.6)	
	Woman	78 (55.3)	69 (50.7)	78 (48.4)	
**Chronically ill (missing n=6), n (%) **					.004^b^
	No or unknown	50 (35.5)	53 (39.3)	84 (53.5)	
	Yes	91 (64.5)	82 (60.7)	73 (46.5)	
**Life status (missing n=2), n (%) **					<.001^b^
	Working	63 (44.7)	61 (44.5)	65 (40.9)	
	Not working^c^	47 (33.3)	30 (21.9)	22 (13.8)	
	Retired	26 (18.4)	42 (30.7)	68 (42.8)	
	Studying	5 (3.5)	4 (2.9)	4 (2.5)	
**Educational level^d^ (missing n=13), n (%) **					.25^b^
	Low	28 (20.0)	31 (23.5)	46 (29.9)	
	Intermediate	51 (36.4)	53 (40.2)	49 (31.8)	
	High	61 (43.6)	48 (36.4)	59 (38.3)	
**Background (missing n=17), n (%) **					.048^b^
	Not native Dutch	12 (8.6)	23 (17.4)	27 (17.9)	
	Dutch	127 (91.4)	109 (82.6)	124 (82.1)	
**Satisfaction with hospital care (missing n=3), n (%) **					.001^b^
	Very dissatisfied	4 (2.8)	3 (2.2)	4 (2.5)	
	Dissatisfied	1 (0.7)	0 (0.0)	0 (0.0)	
	Neutral	5 (3.5)	3 (2.2)	9 (5.6)	
	Satisfied	54 (38.3)	62 (45.9)	84 (52.5)	
	Very satisfied	76 (53.9)	62 (45.9)	48 (30.0)	
	No opinion	1 (0.7)	5 (3.7)	15 (9.4)	

^a^Based on ANOVA.

^b^Based on chi-square.

^c^Unemployed, incapacitated, housewife/houseman.

^d^“Low” indicates no education or low secondary or vocational education; “intermediate” indicates intermediate vocational education or higher general secondary education; and “high” indicates higher vocational education or university.

**Table 2:** Differences between portal users and nonusers.

Characteristics		User (n=141), mean (SD)	Nonuser but aware of portal (n=137), mean (SD)	Nonuser not aware of portal (n=161), mean (SD)	*P* value for differences
**eHeals**					
	Item 1 (missing n=2)	3.85 (0.71)	3.49 (0.93)	3.24 (0.95)	<.001^a^
	Item 2 (missing n=4)	3.89 (0.74)	3.59 (0.88)	3.30 (0.95)	<.001^a^
	Item 3 (missing n=5)	3.97 (0.68)	3.70 (0.84)	3.45 (0.90)	<.001^a^
	Item 4 (missing n=5)	3.85 (0.75)	3.63 (0.87)	3.37 (0.94)	<.001^a^
	Item 6 (missing n=4)	3.89 (0.77)	3.67 (0.89)	3.37 (0.98)	<.001^a^
	Item 7 (missing n=4)	3.74 (0.79)	3.47 (0.93)	3.19 (0.96)	<.001^a^
	Item 8 (missing n=5)	3.55 (0.90)	3.24 (1.02)	3.01 (0.99)	<.001^a^
Modified sum score eHeals^b^ (missing n=7)		26.71 (4.29)	24.81 (5.25)	23.01 (5.53)	<.001^a^
Effort expectancy (missing n=9)		4.11 (0.47)	2.23 (1.23)	N/A^c^	<.001^d^
Facilitating conditions (missing n=10)		3.08 (1.13)	2.14 (1.34)	N/A	<.001^d^
Social influence (missing n=13)		2.24 (1.34)	1.57 (1.27)	N/A	<.001^d^
Performance expectancy (missing n=10)		3.87 (0.80)	1.60 (1.60)	N/A	<.001^d^
Behavioral intention to use (missing n=18)		4.17 (1.09)	3.09 (1.60)	N/A	<.001^d^

^a^Based on ANOVA.

^b^eHealth literacy questionnaire; score per item and sum score of 7 items.

^c^N/A: not applicable.

^d^Based on *t* test.

Satisfaction with the patient portal in total was reported by 84.2% (117/139) of portal users. In total, 3.6% (5/139) of users were dissatisfied with the portal. Between 73.9% and 79.3% of the users were satisfied with the functionalities: treatment reports, results of medical tests, agenda, patient letters (eg, letter from the general practitioner to the medical specialist and vice versa), and patient personal information. Moreover, 43-67.3% of the respondents were satisfied with other parts of the portal (Table 3).

### Predictors of Portal Use

On the basis of the results of the univariate analyses, the following predictors were included in the multivariate model: age, travel time, health situation (chronically ill or not), educational level (high or intermediate), and modified eHealth literacy sum score. As shown in Tables 4 and 5, being chronically ill and eHealth literacy significantly contributed to the multivariate model. The full multivariate model was statistically significant (χ^2^_5_=39.0, *P*=.00), indicating that the model was able to distinguish between portal users and nonusers. Explained variance was between 9% (Cox & Snell R^2^) and 12% (Nagelkerke R^2^), and the model correctly classified 68%.

### Acceptance of the Portal Among Users and Nonusers Aware of the Portal and Prediction of Use

UTAUT constructs were measured in a smaller group of the study population, comprising users and nonusers who were aware of the portal. Mean scores on UTAUT constructs of the portal users were significantly higher than those of the nonusers who were aware of the portal (Table 2). Users more often agreed with factors related to acceptance of the portal when compared with nonusers. In addition, users recorded *not applicable or no opinion* on the acceptance factors less frequently, compared with nonusers who were aware of the portal (Multimedia Appendix 1). Logistic analyses in the part of the population with the significant predictors of the first multivariate model (chronically ill and the modified eHealth literacy sum score) showed that only the modified eHealth literacy sum score (odds ratio, OR 1.09, 95% CI 1.03-1.15) significantly contributed to the model; being chronically ill was not significant (OR 1.17, 95% CI 0.71-1.93).

Univariate analyses in this group showed that all UTAUT constructs had a significant influence on portal use. When we added the UTAUT constructs to the multivariate logistic regression with chronically ill and modified eHealth literacy sum score, it was shown that effort expectancy (OR 13.02, 95% CI 5.68-29.87) and performance expectancy (OR 2.84, 95% CI 1.65-4.90) are significant influencers of portal use. No other variables were statistically significant. The full multivariate model was statistically significant (χ^2^_7_=212.2, *P*=.00), indicating that the model was able to distinguish between portal users and nonusers who were aware of the portal. Explained variance was between 57% (Cox & Snell R^2^) and 76% (Nagelkerke R^2^), and the model correctly classified 89.8%.

**Table 3:** Satisfaction with different parts of the portal.

Satisfaction with different parts of the portal^a^	(Very) dissatisfied, n (%)	Neutral, n (%)	(Very) satisfied, n (%)	No opinion, n (%)
Patient portal in general (n=139)	5 (3.6)	17 (12.2)	117 (84.2)	0 (0.0)
Treatment reports (n=126)	6 (4.8)	24 (19.0)	96 (76.2)	0 (0.0)
Results medical tests (n=129)	7 (5.4)	20 (15.5)	102 (79.1)	0 (0.0)
Agenda (n=119)	3 (2.5)	28 (23.5)	88 (73.9)	0 (0.0)
Patient letters^b^ (n=107)	2 (1.9)	24 (22.4)	81 (75.7)	0 (0.0)
Patient personal information (n=116)	2 (1.7)	22 (19.0)	92 (79.3)	0 (0.0)
Measurements (n=101)	5 (5.0)	28 (27.7)	68 (67.3)	0 (0.0)
e-Consultation (n=88)	4 (5)	33 (38)	51 (58)	0 (0.0)
Medication (n=101)	4 (4.0)	34 (33.7)	63 (62.4)	0 (0.0)
Questionnaires (n=93)	5 (5)	39 (42)	49 (53)	0 (0)
Information (n=97)	6 (6)	29 (30)	62 (64)	0 (0)
E-repeat prescriptions (n=78)	4 (5)	37 (47)	36 (46)	1 (1)
Patients’ personal notes (n=77)	3 (4)	41 (53)	33 (43)	0 (0)

^a^n differs per part of the portal; only parts that were used were scored.

^b^Patient letter is a letter from the general practitioner to the medical specialist and vice versa.

**Table 4:** Predictors of portal use: univariate regression analyses in the total group.

Characteristics	*P* value	Exp (β)	95% CI for Exp (β)
Sex (female)	.25	1.26	0.84-1.89
Age	.01	0.98	0.97-1.00
Travel time	.17	1.00	1.00-1.01
Chronically ill	.02	1.61	1.06-2.44
Life status (working)	.68	1.09	0.73-1.63
Education (high or intermediate)	.12	1.47	0.90-2.40
Modified sum score eHeals	<.001	1.13	1.08-1.19

**Table 5:** Predictors of portal use: multivariate regression analyses in the total group (n=415).

Characteristics	*P* value	Exp (β)	95% CI for Exp (β)
Age	.16	0.99	0.98-1.00
Travel time	.16	1.01	1.00-1.01
Chronically ill	.03	1.62	1.04-2.52
Education (high or intermediate)	.98	1.01	0.58-1.75
Modified sum score eHeals	<.001	1.12	1.07-1.18

## Discussion

### Principal Findings

In a sample of 439 adult patients, we found that 32.1% (141/439) reported to be users of the patient portal; 31.2% (137/439) reported to be a nonuser of the patient portal, but being aware of the existence of the portal; and 36.7% (161/439) were nonusers being unaware of the portal. Most users (117/139, 84.2%) were satisfied with the patient portal and its functionalities. Compared with nonusers, users were statistically younger, less often retired, native Dutch, chronically ill, very satisfied with hospital care, and scored higher on eHealth literacy. Of these factors, being chronically ill and eHealth literate were best predictors of portal use. In a subgroup of patients, users and nonusers who were aware of the portal, the influence of UTAUT constructs was examined. This study showed that effort expectancy and performance expectancy significantly influence portal use in users and nonusers who were aware of the portal, whereas chronically ill and eHealth literacy were not significant predictors.

This study had a large sample size, consisting of patients visiting different departments and accurately reflecting the population of a university hospital. Most patients were willing to participate, which could be explained by the direct and personal approach in waiting rooms. UMCU is one of the first hospitals in the Netherlands to offer a patient portal that provides real-time access to most parts of the medical file and the opportunity of e-consulting to all patients who performed an ID check in the hospital [35]. In other studies, access was often found to be conditional to patient requests or the consent of health care professionals. This should be taken into account when comparing this study’s finding of 32.1% (141/439) patients using the portal with percentages of users in different studies, which varies between 26-51% [36-40]. Besides the way access is provided, other studies also differ in sample size and setting, and the various portals vary in functionalities. For example, a study by Jhamb et al [36] reported a 39% usage rate by patients visiting a nephrology clinic. When looking at this relatively high rate, one should note that patients were invited by staff members to sign up for the portal. A study by Krist et al [37] reported a 26% portal usage rate in a primary care setting in which patients could create an account by themselves. For access to radiology reports, patients voluntarily signed up for the electronic Web portal, leading to 51% usage [38]. In the study of Roelofsen et al [39], 42% of the patients were registered to use a diabetes platform in primary care after they expressed interest in using the resource and were registered by their practice nurse. Of these, 27% subsequently logged on to the platform.

In this study, patient portal use was patient-reported. Often patient log-in is used to measure actual portal use [9]. However, counting log-in incidences does not provide information about functionalities used and lacks contextual information. Another way to report portal use is to distinguish active or passive use [41]—in which active use can be defined as actual communication and interaction, whereas passive use is simply logging in. Similarly, Wallace et al [42] described logging in as viewing, and active use was divided into Web-based requests or services and communication. Moreover, Shimada et al [40] reported about the use of My HealthVet by type 2 diabetes patients and reported that 45.20% used Web-based prescription refills or secure messaging or both, after registration.

In short, providing access after patients’ request could lead to a selection of motivated patients, which biases comparison of use and characteristics of users as well as the percentage of users. Calculating usage based on a subgroup of patients who have the intention to use the portal naturally results in a higher usage percentage than taking the total number of patients who visit a hospital as a whole.

We reported that users differ from nonusers with respect to demographics, being chronically ill, and modified eHealth literacy score. This is consistent with evidence of this study that users, compared with nonusers, are more often younger, female, and highly educated and that patient portals are less often used by minorities [9,43-45]. In addition, users have been found to have higher eHealth literacy levels [9,45,46], probably resulting from their higher levels of education. Furthermore, disease-related characteristics such as being chronically ill or having comorbidities or receiving a greater amount of precious care are linked to higher patient portal usage [37,47,48]. In our multivariate analyses, being chronically ill and modified eHealth literacy score are found to be significant predictors. However, when UTAUT constructs were added to the model in the subgroup of users and nonusers who were aware of the portal, effort expectancy and performance expectancy were shown to be significant predictors. Hence, the likelihood that a patient uses the patient portal increases if a patient perceives the patient portal as beneficial and easy to use and if the patient is skilled and resourceful. These findings are in line with other studies predicting portal use. Emani et al (2012) [18] used the diffusion of innovation model and pointed out that use of a personal health record is influenced by ease of use and relative advantage. Tavares and Oliveira [21] adapted UTAUT to the eHealth consumer context (UTAUT2) and showed that in addition to performance expectancy and effort expectancy, habit and self-perception (defined as perceived severity of the health complaint) are drivers of the intention to use an electronic health record portal [21]. Remarkably, being chronically ill and the modified eHealth literacy score were shown not to be significant predictors when UTAUT constructs were included in the model. Both factors are included in different models to predict portal use, but Tavares and Oliveira were also unable to confirm the influence of being chronically ill [21]. On the other hand, Logue et al revealed that the 5 factors of the Personal Health Records Adoption Mode—personal (including eHealth literacy), environmental, technology, chronic illness, and behavioral factors—influenced adoption of personal health records among the older adults with chronic illness [49,50].

In this study, health care professionals and other important people (eg, family and friends) appear not to play a very influential role in the population sample. This can be explained by the findings of Verstraete et al [35], who showed a relation between a relatively low use by health care professionals in this setting and the fact that the portal was not fully embedded in daily practice. Turvey et al [51] reported that low awareness and lack of knowledge of the patient portal is a barrier to use. Endorsement of a patient portal by health care providers is often shown as an influencing factor [9,52]; so, a more active role of health care professionals as well as integrating the patient portal in usual care [37,53] will increase portal use and in turn will increase the likelihood of positive effects of portal use on health outcomes and efficiency [54,55]. However, the use of patient portals can also give rise to negative outcomes. Real-time access to results of diagnostic tests and medical reports can increase anxiety and worries [35,56,57]. It is therefore important to inform patients about the content of the portal and discuss the options of (temporarily) closing the portal or not logging in to the portal during the diagnostic phase.

### Strengths and Limitations

A number of limitations of this study are worthy of note. First, patients who were not aware of the portal could not answer the UTAUT questions, leading to selective information on users and nonusers who were aware of the portal. Second, this study had a cross-sectional design including adult patients from outpatient departments. Information about parents of children using or not using the patient portal of their child is not included. Moreover, admitted patients were excluded, so it is unknown how often and why a patient portal is used during hospital admission. Furthermore, differences per department were not analyzed because these subgroups were too small and these analyses had not been planned in advance. Third, household income was mentioned earlier as an influencing factor of portal use [36] but was not measured in this study. Generalizability of results is limited because a convenience sample of patients in waiting rooms was included and there was a failure in documenting the total number of patients approached, response rate, and reasons for declining to participate. Finally, the study findings are limited because the validity of the Dutch eHealth literacy questionnaire was considered questionable by Van der Vaart et al [31], and unfortunately, one question was missing on the eHealth literacy scale. We chose to compute the modified sum score of this questionnaire but also reported scores on individual items to be fully transparent.

This study has implications for health care organizations, policy makers, and research. First of all, this study shows that not all patients use the patient portals that are available to them. It is of great importance to realize this and to invest time in educating potential nonusers about the potential benefits of the system. Failure to engage in such promotional-type activities will lead to a failure to maximize usage rate. Until now, patients in the UMCU center have been informed about the portal via paper leaflets and information on the hospital website. During the implementation phase of the patient portal, professionals were instructed to discuss the use of the portal during consultations, but it is known that only half of all professionals did so [35]. Irizarry et al [58] reported that older adults required information about the portal that is targeted at their personal needs and concerns. A recent pilot project in UMCU’s outpatient clinic showed that hosts who provided verbal information tailored to the personal questions of patients about the portal stimulated patients to use the portal. We, therefore, recommend the availability of hosts in waiting rooms. To reach a wider range of patients, we also suggest the inclusion of a (medical) dictionary in the patient portal. This might help to diminish the health literacy gap, as it improves patients’ understanding of complex (medical) language. Further research is needed to investigate whether these add-ons would indeed stimulate patients with lower health literacy and contribute to reducing the information gap. In addition, integration of a patient portal in normal care is necessary to increase awareness of the usefulness of the portal and positive outcomes of the portal and to decrease negative side effects. Health care professionals need to communicate with their patients about the portal and how and when e-consultation and other functionalities could be used. Because UTAUT constructs *effort expectancy* and *performance expectancy* are predictors of portal use, we recommend the involvement of patients in the development and implementation of patient portals. Ryan et al [59] demonstrated that engagement of patients in the early stages of implementation is necessary and that patient-centered partnerships between patients and professionals are needed regarding the use of patient portals. Further research is needed to explore the characteristics of such a partnership from the perspective of patients and professionals.

### Conclusions

To conclude, approximately one-third of the patients of a university hospital self-reported using the patient portal and most of them were satisfied with it. At first sight, being chronically ill and higher scores on the modified eHealth literacy scale were shown to explain portal usage. Including UTAUT constructs in the model showed that effort expectancy, ease of use, knowledge and skills related to portal use, and performance expectancy (perceived usefulness) influenced portal use. Interventions to improve awareness of the portal and eHealth literacy skills of patients and further integration of the patient portal in normal care are needed to increase use and potential benefits for patients.

## Appendix

Multimedia Appendix 1 Factors related to acceptance of the portal for users and nonusers who knew about the portal.

## Abbreviations

ANOVA: analysis of variance
MERC: Medical Ethics Review Committee
OR: odds ratio
TAM: technology acceptance model
UMCU: University Medical Centre Utrecht
UTAUT: Unified Theory of Acceptance and Use of TechnologyMedical Research Involving Human Subjects Act
WMO: Medical Research Involving Human Subjects Act
