# Supplementary material for: Use and the Users of a Patient Portal: Cross-Sectional Study
Source: J Med Internet Res. 2018 Sep 17;20(9):e262. doi: 10.2196/jmir.9418 (PMC6231740; doi:10.2196/jmir.9418)
Supplement: Multimedia Appendix 1 [file jmir_v20i9e262_app1.pdf]

Table 4. Factors related to acceptance of the portal for users and non-user, who knew about the portal (offering as online supplement)

**Factors related to acceptance of the portal under users and non-user, who knew about the portal--**		**User**	**Non user but known with the portal**
Effort Expectancy -a. Log into the portal is easy	(strongly)disagree	9 (6.4%)	8 (6.5%)
	neutral	15 (10.6%)	13 (10.5%)
	(strongly) agree	117 (83.0%)	24 (19.4%)
	not applicable/ no opinion	0 (0.0%)	79 (63.7%)
Effort Expectancy - b. The portal is easy to use	(strongly)disagree	5 (3.5%)	4 (3.2%)
	neutral	13 (9.2%)	14 (11.3%)
	(strongly) agree	123 (87.2%)	23 (18.5%)
	not applicable/ no opinion	0 (0.0%)	83 (66.9%)
Effort Expectancy – c. Information in the portal is understandable	(strongly)disagree	8 (5.7%)	1 (0.8%)
	neutral	23 (16.4%)	14 (11.3%)
	(strongly) agree	109 (77.9%)	21 (16.9%)
	not applicable/ no opinion	0 (0.0%)	88 (71.0%)
Effort Expectancy – d. I have the knowledge that is needed to use the patient portal	(strongly)disagree	1 (0.7%)	11 (8.7%)
	neutral	13 (9.3%)	16 (12.6%)
	(strongly) agree	125 (89.3%)	74 (58.3%)
	not applicable/ no opinion	1 (0.7%)	26 (20.5%)
Effort Expectancy -e. I have the skills that are needed to use the patient portal	(strongly)disagree	0 (0.0%)	5 (3.9%)
	neutral	9 (6.4%)	13 (10.2%)
	(strongly) agree	131 (93.6%)	83 (65.4%)
	not applicable/ no opinion	0 (0.0%)	26 (20.5%)
Effort Expectancy – f. I am physically able to use the portal	(strongly)disagree	1 (0.7%)	2 (1.6%)
	neutral	5 (3.6%)	10 (7.9%)
	(strongly) agree	134 (95.7%)	101 (79.5%)
	not applicable/ no opinion	0 (0.0%)	14 (11.0%)
Social Influence – a.People who are important to me, think that I have to use the portal	(strongly)disagree	11 (8.0%)	17 (13.4%)
	neutral	24 (17.5%)	22 (17.3%)
	(strongly) agree	47 (34.3%)	26 (20.5%)
	not applicable/ no opinion	55 (40.1%)	62 (48.8%)

Social Influence - b. My health care professional stimulated the use of the portal	(strongly)disagree	39 (28.3%)	42 (33.1%)
	neutral	33 (23.9%)	20 (15.7%)
	(strongly) agree	39 (28.3%)	16 (12.6%)
	not applicable/ no opinion	27 (19.6%)	49 (38.6%)
Facilitating Conditions – a. Help is available when I do not know how to use the portal	(strongly)disagree	7 (5.1%)	8 (6.3%)
	neutral	27 (19.7%)	15 (11.8%)
	(strongly) agree	53 (38.7%)	60 (47.2%)
	not applicable/ no opinion	50 (36.5%)	44 (34.6%)
Facilitating Conditions – b. I have sufficient information to work with the portal	(strongly)disagree	13 (9.3%)	27 (21.3%)
	neutral	24 (17.1%)	20 (15.7%)
	(strongly) agree	94 (67.1%)	44 (34.6%)
	not applicable/ no opinion	9 (6.4%)	36 (28.3%)
Facilitating Conditions -c. Information about the patient portal (website, flyer) is clear	(strongly)disagree	6 (4.3%)	2 (1.6%)
	neutral	23 (16.4%)	21 (16.5%)
	(strongly) agree	94 (67.1%)	36 (28.3%)
	not applicable/ no opinion	17 (12.1%)	68 (53.5%)
Performance Expectancy – a. I think that the portal is a useful tool	(strongly)disagree	4 (2.8%)	2 (1.6%)
	neutral	11 (7.8%)	21 (16.5%)
	(strongly) agree	126 (89.4%)	49 (38.6%)
	not applicable/ no opinion	0 (0.0%)	55(43.3%)
Performance Expectancy –b. By using the portal I feel more involved in my care	(strongly)disagree	12 (8.5%)	6 (4.7%)
	neutral	21 (14.9%)	21 (16.5%)
	(strongly) agree	104 (73.8%)	27 (21.3%)
	not applicable/ no opinion	4 (2.8%)	73 (57.5%)
Performance Expectancy – c. By using the portal I feel I have more control over my health	(strongly)disagree	12 (8.6%)	12 (9.5%)
	neutral	41 (29.3%)	21 (16.7%)
	(strongly) agree	82 (58.6%)	16 (12.7%)
	not applicable/ no opinion	5 (3.6%)	77 (61.1%)
Behavioral Intention to Use - Probably, I will use the portal in the future	(strongly)disagree	2 (1.4%)	7 (5.8%)
	neutral	6 (4.3%)	21 (17.4%)
	(strongly) agree	125 (89.9%)	72 (59.5%)
	not applicable/ no opinion	6 (4.3%)	21 (17.4%)
Recommendation - I will recommend the portal to others	(strongly)disagree	4 (2.8%)	8 (6.3%)
	neutral	22 (15.6%)	29 (22.8%)
	(strongly) agree	111 (78.7%)	23 (18.1%)
	not applicable/ no	4 (2.8%)	67 (52.8%)

	opinion		
